# Subcellular mRNA localisation at a glance

**DOI:** 10.1242/jcs.114272

**Published:** 2014-05-15

**Authors:** Richard M. Parton, Alexander Davidson, Ilan Davis, Timothy T. Weil

**Affiliations:** 1 Department of Biochemistry, University of Oxford, South Parks Road, Oxford OX1 3QU, UK; 2 Department of Zoology, University of Cambridge, Downing Street, Cambridge CB2 3EJ, UK

**Keywords:** Cell Biology, Develoment, Polarity, mRNA

## Abstract

mRNA localisation coupled to translational regulation provides an important means of dictating when and where proteins function in a variety of model systems. This mechanism is particularly relevant in polarised or migrating cells. Although many of the models for how this is achieved were first proposed over 20 years ago, some of the molecular details are still poorly understood. Nevertheless, advanced imaging, biochemical and computational approaches have started to shed light on the cis-acting localisation signals and trans-acting factors that dictate the final destination of localised transcripts. In this Cell Science at a Glance article and accompanying poster, we provide an overview of mRNA localisation, from transcription to degradation, focusing on the microtubule-dependent active transport and anchoring mechanism, which we will use to explain the general paradigm. However, it is clear that there are diverse ways in which mRNAs become localised and target protein expression, and we highlight some of the similarities and differences between these mechanisms.

## Introduction

mRNA localisation is a common and conserved means of targeting proteins to their site of function, and is important in a diverse range of cellular and developmental functions (reviewed in Medioni et al., 2012) with clear links to human diseases (Jeibmann et al., 2009).

**Figure:**
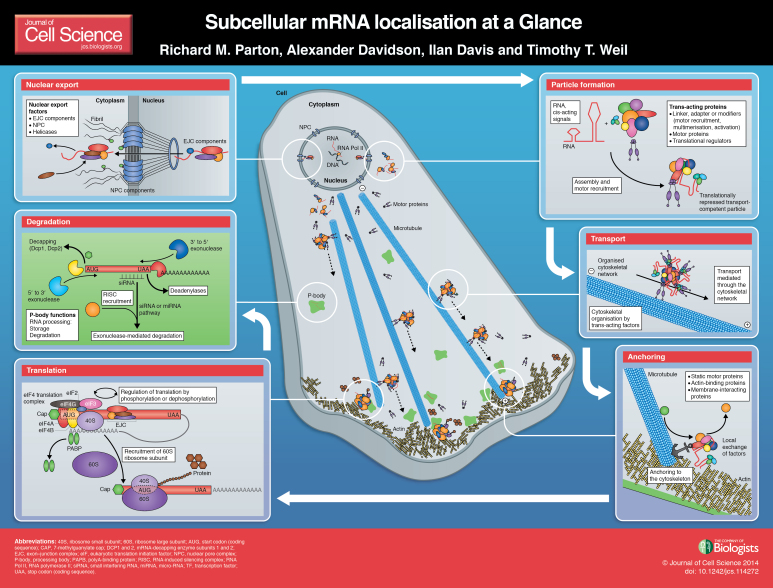


Throughout their journey, mRNA transcripts never exist alone; they bind to a number of proteins to form mRNA–protein complexes, the ribonucleoprotein (RNP) complexes, (Box 1) (reviewed in Kato and Nakamura, 2012). Furthermore, as RNP complexes travel through the cell, there is evidence for dynamic remodeling of these complexes by different trans-acting protein factors that interact with regulatory cis-acting mRNA sequences (Otero et al., 2001). These interactions dictate the fate of mRNAs, so an understanding of these interactions is crucial for explaining the precise spatio-temporal control of localisation and translation. These biological processes have proven challenging to study because of the diversity of proteins and mRNA sequences involved. However, there has recently been significant progress owing to innovative experimental approaches (Box 2).

In this Cell Science at a Glance article and accompanying poster, we focus on localised coding mRNAs in polarised or migrating cells. Using a mechanism of ‘active transport and anchoring’ as a basis for comparison, we follow the fate of mRNA from nuclear export, particle formation (see Box 1), transport, anchoring, translation, to degradation (see poster), highlighting some of the recent exciting findings.

## Nuclear export

mRNAs start their journey in the nucleus where they are transcribed from DNA (Shandilya and Roberts, 2012). Nascent transcripts are spliced, typically co-transcriptionally, to generate the coding mRNA in a process that removes introns, followed by the deposition of the exon–junction complex (EJC) and other components necessary for export and localisation (Baurén and Wieslander, 1994; Natalizio and Wente, 2013; Saulière et al., 2012). At this point, the emerging mRNAs associate, for the first time, with protein co-factors (Box 1) to form RNP complexes (Holt and Bullock, 2009; Natalizio and Wente, 2013).

A growing theme that emerges from the recent literature is that, even at this early stage, the association of localising mRNAs with proteins in the nucleus can dictate their future fate. First, recent work on the EJC suggests that the formation of RNP multimers through interaction with other EJC complexes and with serine- and arginine-rich (SR) proteins plays a role in packaging mRNAs, and in preparing them for nuclear export (Singh et al., 2012). More generally, a host of protein associations is initiated co-transcriptionally in the nucleus and appears to change continually throughout the different stages of mRNA localisation (Marchand et al., 2012; Rodríguez-Navarro and Hurt, 2011; Tran et al., 2007; Trcek et al., 2010). The formation of RNP complexes is directed by specific regions (cis-acting domains), which typically comprise double-stranded secondary structures including hairpins, stem loops and bulges (Amrute-Nayak and Bullock, 2012; Hamilton et al., 2007; Patel et al., 2012; Wilhelm and Vale, 1993). Second, alternative splicing can redefine or shuffle these cis-acting sequences to generate RNP complexes with varying trans-acting protein composition that dictates differential fates of mRNAs (Horne-Badovinac and Bilder, 2008; Trcek et al., 2010). Third, direct interactions of RNP complexes with the nuclear pore complex (NPC) can be essential for the formation of an export- and localisation-competent RNP complex. For example in *Saccharomyces cerevisiae*, mutants for the nucleoporin protein Nup60p, fail to export *ASH1* mRNA from the nucleus and transcripts that are exported do not localise correctly (Powrie et al., 2011).

Once mRNAs are spliced, RNP complexes become export competent through the addition of further co-factors and diffuse through the interchromatin spaces in the nucleoplasm to the NPC (Mor et al., 2010). At the NPC, a conserved and highly complicated structure, they are exported through interactions with co-factors such as nuclear RNA export factor 1 and NTF2-related export protein 1 (NXF1 and NXT1, respectively) and the transcription export complex (TREX), which are essential for nuclear export (Natalizio and Wente, 2013; Rodríguez-Navarro and Hurt, 2011; Valkov et al., 2012). In *Drosophila* embryos and rat myoblasts, the export path has been shown to be random (Politz et al., 2003; Wilkie and Davis, 2001). However, the exact routes taken by RNP complexes remains an area of debate and may indeed be polarised with respect to the cell. Recent breakthroughs achieved by using ‘super registration’ fluorescence microscopy approaches, which allow a direct visualisation of mRNA export in transgenic mouse cell lines (Grünwald and Singer, 2010), show that not all nuclear pores are equally active at the same time. Furthermore, mRNAs were shown to move bidirectionally through the pores, with docking at and release from the nuclear pore being the rate-limiting steps, rather than translocation through the channel. There is also evidence that translocation of a large RNP complex through a channel of limited diameter is facilitated by RNA helicases, which are essential for nuclear export and mRNA quality control (reviewed in Valkov et al., 2012), and can specifically interact with nucleoporins and promote mRNA remodeling (Montpetit et al., 2011).

Interestingly, export through the NPC is not the only path to the cytoplasm. Recently, an alternative mechanism has been discovered in the *Drosophila* neuromuscular junction where large RNP complexes exit the nucleus through vesicular budding (Jokhi et al., 2013; Speese et al., 2012), a process thought to be utilized by some DNA viruses. Regardless of the underlying mechanism, once the mRNA has arrived at the cytoplasmic face of the nucleus, it is ready for the next phase of its journey.

## Transport and anchoring

Early studies of mRNA transport in the cytoplasm focused on the overall localisation of transcripts through the visualisation of endogenous mRNA by *in situ* hybridisation (Jeffery et al., 1983; Lawrence and Singer, 1986; Weil et al., 2010a). The subsequent development of the MS2 bacteriophage RNA stem loop bound by MS2 coat protein fusion to a fluorescent protein (MS2-MCP system) and recent imaging advances (see Box 2) combined with the use of mutants that affect transacting protein factors, motors and the cytoskeleton, and also drug treatments, showed that different mRNAs localise by means of different mechanisms (Bertrand et al., 1998; Forrest et al., 2003; Jaramillo et al., 2008; Lerit and Gavis, 2011; Medioni et al., 2012; Takatori et al., 2010). For example, in late *Drosophila* oogenesis, *bicoid* (*bcd*) is localised by active transport and *nanos* (*nos*) is localised by means of diffusion and trapping (Forrest et al., 2003; Weil et al., 2006). Biochemical experiments have supported and expanded our understanding of the trans-acting factors that are required for the regulation of these processes (McDermott et al., 2012; Müller et al., 2011; Snee et al., 2005). The classic view of RNP complexes is that they are composed of higher order mRNA complexes. This certainly appears to be the case for *oskar* (*osk*) mRNA, where multiple mRNA particles associate with each other to form large transport particles or granules (Chekulaeva et al., 2006; Kato and Nakamura, 2012). However, recent research following individual mRNAs indicates that oligomerisation is not obligatory for transport (Amrute-Nayak and Bullock, 2012). In this section, we focus primarily on the current understanding of mRNA localisation along microtubules by means of active motor-dependent transport in polarised cells. This is exemplified by the classic axis-determining mRNAs in *Drosophila* that move on microtubule tracks, with kinesin transporting *osk* mRNA in the plus-end direction and dynein driving *gurken* (*grk*) mRNA transport to the microtubule minus end (MacDougall et al., 2003; Zimyanin et al., 2008).

An essential requirement for localisation is the linkage of the correct motor to the cargo and the connection of this complex to the cytoskeleton. Bicaudal-D (BicD) and Egalitarian (Egl) are two linkers with key roles in axis determination in *Drosophila* oocytes and embryos (Mach and Lehmann, 1997; Bullock and Ish-Horowicz, 2001; Navarro et al., 2004). Egl directly binds to different cis-acting elements that mediate mRNA localisation and BicD regulates the linkage of mRNA cargo to dynein. A recent study provides insight into the molecular mechanism of motor protein recruitment by solving the crystal structure of the cargo-binding domain of BicD (Liu et al., 2013), which shows that cargo binds to the homotypic domain of BicD and releases the auto inhibition of its heterotypic coiled-coil domain, thus allowing this domain to bind to dynein.

Work *in vitro* has suggested that the number of motors that are recruited to an RNP complex can be controlled by cis-acting localisation elements in the mRNA (Amrute-Nayak and Bullock, 2012). Similarly, recruitment of multiple copies of the class V myosin Myo4 by She2 has been shown to enhance transport of *ASH1* mRNA along actin in *S. cerevisiae* (Chung and Takizawa, 2010). Additional levels of regulating transport efficiency also exist, for instance mediated by proteins such as Pat1, which interacts with cargo adaptors to regulate the motility of kinesin heavy chain on microtubules and is necessary for *osk* mRNA localisation (Loiseau et al., 2010). The microtubule-associated protein (MAP) ensconsin interacts with microtubules and kinesin-1 to increase efficiency of motor recruitment (Sung et al., 2008). This is different to typical MAPs, which affect cytoskeletal stability and organisation.

The classic model of mRNA localisation poses that it is underpinned by a highly polarised cytoskeletal network (Clark et al., 1994). This model predicts that mRNAs exhibit a concerted directional motion. However, research on the dynamics of RNP complexes often shows a non-uniform rather than continuous, processive movement (Sinsimer et al., 2013). One explanation for this is that the dynein motor is capable of reversing direction (Gross, 2004). Another explanation comes from recent *in vivo* and *in vitro* analysis that has shown that individual mRNA cargos can undergo bidirectional movement owing to transport by multiple motors. In the case of *Vg1* mRNA in frogs, there are different phases of transport during localisation. An initial highly unidirectional dynein-dependent phase is followed by multiple rounds of bidirectional transport for which kinesin-1, kinesin-2 and dynein are required, before transcripts are anchored at the vegetal cortex (Gagnon et al., 2013).

In *Drosophila* oocytes, live imaging of *osk* mRNA and visualisation of microtubules using green fluorescent protein (GFP) tagged to end-binding protein 1 (EB1) revealed an apparently random organisation with subtle bias towards the posterior, rather than the expected highly polarised cytoskeletal network (Parton et al., 2011; Zimyanin et al., 2008). Additional control of transport can be mediated through the regulation of the properties of the tracks themselves (Gardner et al., 2011), such as regulating the extension and catastrophic collapse of microtubules.

Once the mRNA has reached its destination, anchoring is a common mechanism for maintaining mRNA localisation. For example *grk* mRNA is anchored by dynein at the *Drosophila* oocyte dorsal anterior corner, whereas *nos* mRNA that is diffusing in the ooplasm is trapped by actin at the posterior pole (Delanoue et al., 2007; Forrest et al., 2003). In *Xenopus*, *Vg1* mRNA is maintained at the vegetal pole of the oocyte by the actin cytoskeleton (Yisraeli et al., 1990).

Actin has also been implicated in the organisation and function of the microtubule cytoskeleton and has been demonstrated to be of particular importance for the localisation of *bicoid* mRNA (Weil et al., 2010b) and in organising a polarised microtubule cytoskeleton in the *Drosophila* oocyte (Dahlgaard et al., 2007). Actin can also function as a track on which cargoes can be transported. Here, actin not only acts in the short-range localisation of mRNAs, as is the case for *ASH1* mRNA in yeast (Bertrand et al., 1998), but also in long-range vesicle transport in mouse oocytes (Schuh et al., 2011; reviewed in St Johnston, 2005). Together, these examples demonstrate that there is no clear universal mechanism for mRNA transport. The future challenge is to understand to what extent the differential localisation of mRNA species is achieved through distinct transport mechanisms that operate in parallel.

## Translation

For effective localised protein expression, mRNAs are kept translationally silent during transport and are activated for translation when they are anchored at their destination. Cis-acting mRNA sequences are almost certainly responsible for both the translational repression of mRNAs and their activation by directing their interaction with trans-acting proteins. Several strategies have been proposed, including the binding of factors that block or mask the interaction of translational activators, either by sequestering these translation activation factors or by occupying their interaction sites on the mRNA. These factors could either physically restrict the access of the translational machinery or act by regulating the length of the poly(A)-tail on the transcripts, whose extension is known to precede translational activation (Rosenthal et al., 1983).

One of the best-studied mechanisms of translational repression involves the eukaryotic translation initiation factor 4E (eIF4E) pathway (Jackson et al., 2010). In this repression pathway, mRNAs are prevented from initiating translation that requires the recruitment of the 40S ribosomal subunit through assembly of eIF4F (eIF4G+eIF4E and eIF4A) at the 5′ cap. Translational repressors can bind directly to the mRNA, thus masking the sites for eIF4E binding. For example, the *Drosophila* protein Bruno interacts with Bruno-response elements on mRNA, thus blocking initiation of translation (Kim-Ha et al., 1995). Alternatively, factors such as the *Drosophila* ovarian protein Cup can bind eIF4E and prevent it from accessing mRNA, thereby inhibiting initiation of translation (Chekulaeva et al., 2006; Nakamura et al., 2004; Piccioni et al., 2005; Wilhelm et al., 2003). Overexpression of eIF4E has been shown to lead to autism-like behavior in mice (Santini et al., 2013), and HIV-1 was shown to be able to maintain virus-specific protein synthesis when eIF4E is downregulated (Sharma et al., 2012). This demonstrates that eIF4E-mediated translational repression is important in a range of cells and circumstances.

A further well-characterised means of controlling the level of proteins synthesis from mRNA is through polyadenylation or deadenylation. In frogs and flies, there are many examples of cytoplasmic polyadenylation elements (CPEs) that reside in the 3′ UTR of mRNA, which are bound by the CPE-binding protein (CPEB) to control translation (Chang et al., 1999; Christerson and McKearin, 1994; Hake and Richter, 1994; Radford et al., 2008). Recently, work in the hippocampus of mice has shown that mutants in which the translational repressor of poly(A)-binding protein (PABP) has been knocked out show an increased translation of Ca^2+^/calmodulin-dependent protein kinase II alpha (*Camk2a*) mRNA, a factor involved in many signaling cascades that are regulated by Ca^2+^. Translational activation through the release of PABP-dependent repression has been demonstrated following electrode stimulation and is important for synaptic plasticity and learning (Khoutorsky et al., 2013).

Mouse models have also highlighted the complexity of translational regulation and revealed new means by which kinase activity regulates mRNA translation. For instance, the mouse protein kinase R (PKR)-like endoplasmic reticulum kinase (PERK, also known as EIF2AK3) has been shown to have a key role in brain function by phosphorylating eIF2α (also known as EIF2A), a key regulator of translational activity (Trinh et al., 2012). A unique level of intricacy has been shown to be provided by the interaction of fragile X mental retardation 1 (FMRP, also known as FMR1) protein and the human topoisomerase 3-beta-1 (Top3β, also known as TOP3B) (Xu et al., 2013), as this complex appears to regulate multiple mRNAs in neurons and – in Top3β mutants – there is a reduction in the expression of genes within the neuromuscular junction (NMJ) that are important for neural function. In addition to FMRP, other RNA-binding proteins found in the *Drosophila* nervous system, such as Syncrip (Syp) and Staufen (Stau), have distinct roles in regulation mRNA translation in other stages of development (McDermott et al., 2012; Barbee et al., 2006).

Although non-coding RNAs are thought to primarily have a role in mRNA degradation (see following section), an additional role is emerging in translational regulation. In flies, a reversible mechanism for regulating gene expression through the pathway via microRNA (miRNA) and argonaute 2 (AGO2) (Muddashetty et al., 2011) involves the phosphorylation of *Drosophila* FMR1 at the synapse. The direct targeting of precursor miRNA – a long piece of double-stranded RNA (dsRNA) from which mature miRNAs are generated – to neuronal dendrites has been shown to be mediated by the DEAH-box helicase 36 (DHX36), suggesting that the localisation of precursor miRNA is an important plasticity mechanism (Bicker et al., 2013). Interestingly, an miRNA-independent mechanism for the recruitment of Argonaute-1 (AGO1) to nanos (*nos*) mRNA in the *Drosophila* early embryo has been described that acts through the smaug protein, suggesting that there are different mechanisms of translational regulation that most likely work in concert (Pinder and Smibert, 2013).

The recent characterisation of protein components that are involved in translational repression of mRNAs has revealed that the subcellular organisation of protein complexes contributes to efficient translational regulation (Balagopal and Parker, 2009). Processing bodies (P-bodies) are known locations of mRNA translational control and degradation and have, therefore, been referred to as hubs for RNA metabolism in yeast (Aizer et al., 2008; reviewed in Balagopal and Parker, 2009). It has recently been shown that they are also important in regulating developmentally relevant transcripts. We have also shown that it is important where exactly in P-bodies mRNAs localise because the inside of electron-dense P-bodies does not support translation, whereas their localisation at the P-body edge allows translation to occur (Weil et al., 2012). Other work from yeast suggests that P-bodies are also the place where mRNAs terminate their journey through the cell (Aizer et al., 2008; Brengues et al., 2005).

## Degradation

The tight regulation of mRNAs in both space and time ultimately requires mRNA degradation. Although there are several pathways of degradation, the most common is through 5′ to 3′ exonuclease activity, following deadenylation and decapping (reviewed in Decker and Parker, 2012). In yeast, this process is linked with cytoplasmic P-bodies, which are associated with translationally repressed transcripts (Teixeira et al., 2005). These are distinct from stress granules, related cytoplasmic foci that have a similar composition and are likely to share functions. Stress granules assemble under physiological conditions when translation is stalled rather than repressed (Buchan and Parker, 2009). Interestingly, recent research indicates that mRNA can be degraded in a 5′ to 3′ fashion as translation is occurring (Hu et al., 2009), suggesting that the removal of ribosomes and the activity of exonucleases are not necessarily sequential events.

In *Drosophila*, P-body proteins have been found in neurons and oocytes, and have been suggested to be important for regulating transcripts (Barbee et al., 2006; Weil et al., 2012). In the case of *grk* mRNA, these bodies may be involved in regulating the level of transcripts and act as a dosage and temporal control, although degradation was not explicitly demonstrated in our study (Weil et al., 2012). An additional mechanism of mRNA degradation that is relevant for the control of spatially and temporally constrained transcripts is miRNA-mediated degradation. In the case of the maternal to zygotic transition in zebrafish and *Drosophila* embryos, maternal mRNAs are cleared from the embryo by miRNA-mediated deadenylation (Bushati et al., 2008; Giraldez et al., 2006).

Another mechanism of mRNA regulation is at the level of the DNA sequence, which can affect mRNA levels through the control of decay rates. Recent evidence from yeast shows that DNA promoter elements can affect the decay kinetics of their respective mRNAs after nuclear export (Bregman et al., 2011).

The specific mechanisms that regulate the degradation of mRNA transcripts after their final localisation have yet to be extensively studied and, thus, the role of P-bodies as sites of degradation remains to be established, mainly owing to the difficulty in characterising these labile bodies and in observing their interactions with mRNA.

## Perspectives

New imaging technologies, bioinformatics and biochemistry have facilitated major advances in our understanding of the composition, motility and translational regulation of RNP complexes. One successful example of these applications is in the analysis of RNP complex composition of nascent transcripts as they are being processed during splicing in the nucleus and at the NPC.

However, many important questions in the field remain unanswered, such as what is the extent of RNP complex remodeling in the cytoplasm and how is it regulated? How prevalent is the function of small non-coding RNA in the translational regulation of mRNAs? Do the numerous regulatory mechanisms facilitate the localisation of diverse transcripts or are they redundant mechanisms to protect crucial biological processes? The challenge will be to place the biochemical information with regard to the components that are involved in the context of *in vivo* data to fully understand the molecular mechanism of mRNA localisation with respect to cell and tissue function.

**Box 1. Particle formation**

Biochemical and genetic studies have shown how, for localising mRNAs, particular proteins determine each step of their journey (reviewed in Kato and Nakamura, 2012). Although a diverse array of proteins are involved, these trans-acting factors can be grouped into several classes (see poster).

Understanding the interaction between cis- and trans-acting components, RNP complex composition and remodeling are crucial for understanding the molecular mechanisms underlying mRNA localisation (Otero et al., 2001). Assembly of the multiple proteins required for regulating the translational state of the mRNA has been shown to involve numerous sites of weak interaction and involve modification of the mRNA folding (Chao et al., 2010; St Johnston, 2005). One of the intriguing questions is how the composition of the RNP complexes confers the specific fates of different mRNAs. Although there is evidence that different mRNAs with the same destination are packaged and transported together in the same RNP complex (Lange et al., 2008), it was hypothesised that particles with distinct destinations can be distinguished by certain trans-acting factors that are specific for the particular destination (Cha et al., 2001). It has been difficult to confirm this hypothesis because many mRNA binding proteins associate with a wide range of different transcripts; for example, the highly conserved double-stranded RNA (dsRNA)-binding protein Staufen (Stau) associates with both *oskar (osk)* and *bicoid (bcd)* mRNA in *Drosophila* oocytes (Ephrussi et al., 1991; St Johnston et al., 1989). This notion is further complicated by evidence from biochemical analysis such as the ‘atlas’ of mammalian mRNA-interacting proteins, which has revealed the enormous diversity of trans-acting factors (Castello et al., 2012).

These complex data suggest an alternative view of the specificity of RNP complexes, in that their flexibility in regulating transcripts is based on an unique complement of overlapping cofactors for each mRNA species (Castello et al., 2012; Castello et al., 2013; Kato and Nakamura, 2012; McDermott et al., 2012). A major remaining question is what precise combination of factors is required for the correct localization and translational regulation of each mRNA species, and how this is modified during the life cycle of the transcripts.

**Box 2. Imaging techniques to study mRNA localisation**

Early studies of mRNA localisation were reliant upon *in situ* hybridisation in fixed material, a slow and laborious method. Recent advances in fluorescent *in situ* hybridisation (FISH) now allow the rapid labeling of single mRNA molecules with high sensitivity and selectivity (Buxbaum et al., 2014; Little et al., 2011). FISH has also been applied to live cells (Santangelo et al., 2009). However, despite these improvements, *in situ* hybridisation remains limited in its application to certain tissues. An alternative approach to label mRNA in live cells is through injection of *in vitro* synthesized RNA that incorporates fluorescent dyes. By using this technique, it has been possible to rapidly screen a range of mutated RNAs to determine the crucial cis-acting sequences that determine mRNA localisation in *Drosophila* embryos and oocytes (Bullock and Ish-Horowicz, 2001; Van De Bor et al., 2005).

A breakthrough in live imaging that also emerges as one of the most useful approaches for *in vivo* study of mRNA localisation is the MS2 bacteriophage RNA stem loop bound by MS2 coat protein fusion to a fluorescent protein (MS2-MCP system). It was originally developed in yeast (Bertrand et al., 1998) but has since been extended to several organisms, including *Drosophila* (Forrest et al., 2003), *Mus musculus* (Park et al., 2014), *Xenopus* (Gagnon et al., 2013) and plants (Hamada et al., 2003). It has also been used to detect nascent mRNA as it is transcribed (Hocine et al., 2013; Yunger et al., 2013). Improvements are directed towards maximising the detection of mRNAs, such as by increasing the number and optimisation of MS2 loops (Lionnet et al., 2011). An alternative construct uses the bacteriophage PP7 and also involves the interaction between a stem loop and a viral coat protein (Wu et al., 2012). Using the MS2 and PP7 systems in combination might allow the tracking of two different species of mRNAs simultaneously and could provide insights into how transcripts segregate.

However, one limitation of the MS2 and PP7 systems is the potential lack of contrast caused by unbound MCP. This may be overcome by targeting MCP to the nucleus (Bertrand et al., 1998), although this would compromise the ability to follow mRNAs in and around the nucleus. Recently, a combination of MS2 and PP7 has been described that drives a highly specific split-fluorescent protein complementation on the mRNA and allows for high-contrast labeling of individual transcripts (Hocine et al., 2013).

Furthermore, different tissues present different challenges for imaging approaches, which could be addressed by using advances in imaging techniques. For instance, fast, sensitive widefield microscopy is useful when imaging fast-moving mRNA particles (Zimyanin et al., 2008), whereas when imaging within thick samples, such as *Drosophila* embryos, multi-photon microscopy can be useful (Sinsimer et al., 2013). Total internal reflection microscopy (TIRF) is ideal for *in vitro* applications because of its high axial resolution (Amrute-Nayak and Bullock, 2012), whereas 3D structured illumination microscopy (3D-SIM) gives axial and lateral super-resolution while being flexible enough to be applied when investigating conventionally prepared materials (Weil et al., 2010a).

## Supplementary Material

Poster Panels

## References

[b1] Aizer A. Brody Y. Ler L. W. Sonenberg N. Singer R. H. Shav-Tal Y. (2008). The dynamics of mammalian P body transport, assembly, and disassembly in vivo. Mol. Biol. Cell 19, 4154–4166 10.1091/mbc.E08--05--0513 18653466 PMC2555939

[b2] Amrute-Nayak M. Bullock S. L. (2012). Single-molecule assays reveal that RNA localization signals regulate dynein-dynactin copy number on individual transcript cargoes. Nat. Cell Biol. 14, 416–423 10.1038/ncb2446 22366687 PMC3343632

[b3] Balagopal V. Parker R. (2009). Polysomes, P bodies and stress granules: states and fates of eukaryotic mRNAs. Curr. Opin. Cell Biol. 21, 403–408 10.1016/j.ceb.2009.03.005 19394210 PMC2740377

[b4] Barbee S. A. Estes P. S. Cziko A-M. Hillebrand J. Luedeman R. A. Coller J. M. Johnson N. Howlett I. C. Geng C. Ueda R. (2006). Staufen- and FMRP-containing neuronal RNPs are structurally and functionally related to somatic P bodies. Neuron 52, 997–1009 10.1016/j.neuron.2006.10.028 17178403 PMC1955741

[b5] Baurén G. Wieslander L. (1994). Splicing of Balbiani ring 1 gene pre-mRNA occurs simultaneously with transcription. Cell 76, 183–192 10.1016/0092--8674(94)90182--1 8287477

[b6] Bertrand E. Chartrand P. Schaefer M. Shenoy S. M. Singer R. H. Long R. M. (1998). Localization of ASH1 mRNA particles in living yeast. Mol. Cell 2, 437–445 10.1016/S1097--2765(00)80143--4 9809065

[b7] Bicker S. Khudayberdiev S. Weiß K. Zocher K. Baumeister S. Schratt G. (2013). The DEAH-box helicase DHX36 mediates dendritic localization of the neuronal precursor-microRNA-134. Genes Dev. 27, 991–996 10.1101/gad.211243.112 23651854 PMC3656329

[b8] Bregman A. Avraham-Kelbert M. Barkai O. Duek L. Guterman A. Choder M. (2011). Promoter elements regulate cytoplasmic mRNA decay. Cell 147, 1473–1483 10.1016/j.cell.2011.12.005 22196725

[b9] Brengues M. Teixeira D. Parker R. (2005). Movement of eukaryotic mRNAs between polysomes and cytoplasmic processing bodies. Science 310, 486–489 10.1126/science.1115791 16141371 PMC1863069

[b10] Buchan J. R. Parker R. (2009). Eukaryotic stress granules: the ins and outs of translation. Mol. Cell 36, 932–941 10.1016/j.molcel.2009.11.020 20064460 PMC2813218

[b11] Bullock S. L. Ish-Horowicz D. (2001). Conserved signals and machinery for RNA transport in Drosophila oogenesis and embryogenesis. Nature 414, 611–616 10.1038/414611a 11740552

[b12] Bushati N. Stark A. Brennecke J. Cohen S. M. (2008). Temporal reciprocity of miRNAs and their targets during the maternal-to-zygotic transition in Drosophila. Curr. Biol. 18, 501–506 10.1016/j.cub.2008.02.081 18394895

[b13] Buxbaum A. R. Wu B. Singer R. H. (2014). Single β-actin mRNA detection in neurons reveals a mechanism for regulating its translatability. Science 343, 419–422 10.1126/science.1242939 24458642 PMC4121734

[b14] Castello A. Fischer B. Eichelbaum K. Horos R. Beckmann B. M. Strein C. Davey N. E. Humphreys D. T. Preiss T. Steinmetz L. M. (2012). Insights into RNA biology from an atlas of mammalian mRNA-binding proteins. Cell 149, 1393–1406 10.1016/j.cell.2012.04.031 22658674

[b15] Castello A. Horos R. Strein C. Fischer B. Eichelbaum K. Steinmetz L. M. Krijgsveld J. Hentze M. W. (2013). System-wide identification of RNA-binding proteins by interactome capture. Nat. Protoc. 8, 491–500 10.1038/nprot.2013.020 23411631

[b16] Cha B. J. Koppetsch B. S. Theurkauf W. E. (2001). In vivo analysis of Drosophila bicoid mRNA localization reveals a novel microtubule-dependent axis specification pathway. Cell 106, 35–46 10.1016/S0092--8674(01)00419--6 11461700

[b17] Chang J. S. Tan L. Schedl P. (1999). The Drosophila CPEB homolog, orb, is required for oskar protein expression in oocytes. Dev. Biol. 215, 91–106 10.1006/dbio.1999.9444 10525352

[b18] Chao J. A. Patskovsky Y. Patel V. Levy M. Almo S. C. Singer R. H. (2010). ZBP1 recognition of beta-actin zipcode induces RNA looping. Genes Dev. 24, 148–158 10.1101/gad.1862910 20080952 PMC2807350

[b19] Chekulaeva M. Hentze M. W. Ephrussi A. (2006). Bruno acts as a dual repressor of oskar translation, promoting mRNA oligomerization and formation of silencing particles. Cell 124, 521–533 10.1016/j.cell.2006.01.031 16469699

[b20] Christerson L. B. McKearin D. M. (1994). orb is required for anteroposterior and dorsoventral patterning during Drosophila oogenesis. Genes Dev. 8, 614–628 10.1101/gad.8.5.614 7926753

[b21] Chung S. Takizawa P. A. (2010). Multiple Myo4 motors enhance ASH1 mRNA transport in Saccharomyces cerevisiae. J. Cell Biol. 189, 755–767 10.1083/jcb.200912011 20457760 PMC2872910

[b22] Clark I. Giniger E. Ruohola-Baker H. Jan L. Y. Jan Y. N. (1994). Transient posterior localization of a kinesin fusion protein reflects anteroposterior polarity of the Drosophila oocyte. Curr. Biol. 4, 289–300 10.1016/S0960--9822(00)00068--3 7922338

[b23] Dahlgaard K. Raposo A. A. S. F. Niccoli T. St Johnston D. (2007). Capu and Spire assemble a cytoplasmic actin mesh that maintains microtubule organization in the Drosophila oocyte. Dev. Cell 13, 539–553 10.1016/j.devcel.2007.09.003 17925229 PMC2034408

[b24] Decker C. J. Parker R. (2012). P-bodies and stress granules: possible roles in the control of translation and mRNA degradation. Cold Spring Harb. Perspect. Biol. 4, a012286 10.1101/cshperspect.a012286 22763747 PMC3428773

[b25] Delanoue R. Herpers B. Soetaert J. Davis I. Rabouille C. (2007). Drosophila Squid/hnRNP helps Dynein switch from a gurken mRNA transport motor to an ultrastructural static anchor in sponge bodies. Dev. Cell 13, 523–538 10.1016/j.devcel.2007.08.022 17925228

[b26] Ephrussi A. Dickinson L. K. Lehmann R. (1991). Oskar organizes the germ plasm and directs localization of the posterior determinant nanos. Cell 66, 37–50 10.1016/0092--8674(91)90137--N 2070417

[b27] Forrest K. M. Gavis E. R. (2003). Live imaging of endogenous RNA reveals a diffusion and entrapment mechanism for nanos mRNA localization in Drosophila. Curr. Biol. 13, 1159–1168 10.1016/S0960--9822(03)00451--2 12867026

[b28] Gagnon J. A. Kreiling J. A. Powrie E. A. Wood T. R. Mowry K. L. (2013). Directional transport is mediated by a Dynein-dependent step in an RNA localization pathway. PLoS Biol. 11, e1001551 10.1371/journal.pbio.1001551 23637574 PMC3640089

[b29] Gardner M. K. Zanic M. Gell C. Bormuth V. Howard J. (2011). Depolymerizing kinesins Kip3 and MCAK shape cellular microtubule architecture by differential control of catastrophe. Cell 147, 1092–1103 10.1016/j.cell.2011.10.037 22118464

[b30] Giraldez A. J. Mishima Y. Rihel J. Grocock R. J. Van Dongen S. Inoue K. Enright A. J. Schier A. F. (2006). Zebrafish MiR-430 promotes deadenylation and clearance of maternal mRNAs. Science 312, 75–79 10.1126/science.1122689 16484454

[b31] Gross S. P. (2004). Hither and yon: a review of bi-directional microtubule-based transport. Phys. Biol. 1, R1–R11 10.1088/1478--3967/1/2/R01 16204815

[b32] Grünwald D. Singer R. H. (2010). In vivo imaging of labelled endogenous β-actin mRNA during nucleocytoplasmic transport. Nature 467, 604–607 10.1038/nature09438 20844488 PMC3005609

[b33] Hake L. E. Richter J. D. (1994). CPEB is a specificity factor that mediates cytoplasmic polyadenylation during Xenopus oocyte maturation. Cell 79, 617–627 10.1016/0092--8674(94)90547--9 7954828

[b34] Hamada S. Ishiyama K. Choi S-B. Wang C. Singh S. Kawai N. Franceschi V. R. Okita T. W. (2003). The transport of prolamine RNAs to prolamine protein bodies in living rice endosperm cells. Plant Cell 15, 2253–2264 10.1105/tpc.013466 14508010 PMC197292

[b35] Hamilton R. S. Davis I. (2007). RNA localization signals: deciphering the message with bioinformatics. Semin. Cell Dev. Biol. 18, 178–185 10.1016/j.semcdb.2007.02.001 17452113 PMC3082379

[b36] Hocine S. Raymond P. Zenklusen D. Chao J. A. Singer R. H. (2013). Single-molecule analysis of gene expression using two-color RNA labeling in live yeast. Nat. Methods 10, 119–121 10.1038/nmeth.2305 23263691 PMC3899799

[b37] Holt C. E. Bullock S. L. (2009). Subcellular mRNA localization in animal cells and why it matters. Science 326, 1212–1216 10.1126/science.1176488 19965463 PMC3785123

[b38] Horne-Badovinac S. Bilder D. (2008). Dynein regulates epithelial polarity and the apical localization of stardust A mRNA. PLoS Genet. 4, e8 10.1371/journal.pgen.0040008 18208331 PMC2213700

[b39] Hu W. Sweet T. J. Chamnongpol S. Baker K. E. Coller J. (2009). Co-translational mRNA decay in Saccharomyces cerevisiae. Nature 461, 225–229 10.1038/nature08265 19701183 PMC2745705

[b40] Jackson R. J. Hellen C. U. T. Pestova T. V. (2010). The mechanism of eukaryotic translation initiation and principles of its regulation. Nat. Rev. Mol. Cell Biol. 11, 113–127 10.1038/nrm2838 20094052 PMC4461372

[b41] Jaramillo A. M. Weil T. T. Goodhouse J. Gavis E. R. Schupbach T. (2008). The dynamics of fluorescently labeled endogenous gurken mRNA in Drosophila. J. Cell Sci. 121, 887–894 10.1242/jcs.019091 18303053 PMC2327291

[b42] Jeffery W. R. Tomlinson C. R. Brodeur R. D. (1983). Localization of actin messenger RNA during early ascidian development. Dev. Biol. 99, 408–417 10.1016/0012--1606(83)90290--7 6194032

[b43] Jeibmann A. Paulus W. (2009). Drosophila melanogaster as a model organism of brain diseases. Int. J. Mol. Sci. 10, 407–440 10.3390/ijms10020407 19333415 PMC2660653

[b44] Jokhi V. Ashley J. Nunnari J. Noma A. Ito N. Wakabayashi-Ito N. Moore M. J. Budnik V. (2013). Torsin mediates primary envelopment of large ribonucleoprotein granules at the nuclear envelope. Cell Rep 3, 988–995 10.1016/j.celrep.2013.03.015 23583177 PMC3683601

[b45] Kato Y. Nakamura A. (2012). Roles of cytoplasmic RNP granules in intracellular RNA localization and translational control in the Drosophila oocyte. Dev. Growth Differ. 54, 19–31 10.1111/j.1440--169X.2011.01314.x 22111938

[b46] Khoutorsky A. Yanagiya A. Gkogkas C. G. Fabian M. R. Prager-Khoutorsky M. Cao R. Gamache K. Bouthiette F. Parsyan A. Sorge R. E. (2013). Control of synaptic plasticity and memory via suppression of poly(A)-binding protein. Neuron 78, 298–311 10.1016/j.neuron.2013.02.025 23622065

[b47] Kim-Ha J. Kerr K. Macdonald P. M. (1995). Translational regulation of oskar mRNA by bruno, an ovarian RNA-binding protein, is essential. Cell 81, 403–412 10.1016/0092--8674(95)90393--3 7736592

[b48] Lange S. Katayama Y. Schmid M. Burkacky O. Bräuchle C. Lamb D. C. Jansen R-P. (2008). Simultaneous transport of different localized mRNA species revealed by live-cell imaging. Traffic 9, 1256–1267 10.1111/j.1600--0854.2008.00763.x 18485054

[b49] Lawrence J. B. Singer R. H. (1986). Intracellular localization of messenger RNAs for cytoskeletal proteins. Cell 45, 407–415 10.1016/0092--8674(86)90326--0 3698103

[b50] Lerit D. A. Gavis E. R. (2011). Transport of germ plasm on astral microtubules directs germ cell development in Drosophila. Curr. Biol. 21, 439–448 10.1016/j.cub.2011.01.073 21376599 PMC3062663

[b51] Lionnet T. Czaplinski K. Darzacq X. Shav-Tal Y. Wells A. L. Chao J. A. Park H. Y. de Turris V. Lopez-Jones M. Singer R. H. (2011). A transgenic mouse for in vivo detection of endogenous labeled mRNA. Nat. Methods 8, 165–170 10.1038/nmeth.1551 21240280 PMC3076588

[b52] Little S. C. Tkačik G. Kneeland T. B. Wieschaus E. F. Gregor T. (2011). The formation of the Bicoid morphogen gradient requires protein movement from anteriorly localized mRNA. PLoS Biol. 9, e1000596 10.1371/journal.pbio.1000596 21390295 PMC3046954

[b53] Liu Y. Salter H. K. Holding A. N. Johnson C. M. Stephens E. Lukavsky P. J. Walshaw J. Bullock S. L. (2013). Bicaudal-D uses a parallel, homodimeric coiled coil with heterotypic registry to coordinate recruitment of cargos to dynein. Genes Dev. 27, 1233–1246 10.1101/gad.212381.112 23723415 PMC3690397

[b54] Loiseau P. Davies T. Williams L. S. Mishima M. Palacios I. M. (2010). Drosophila PAT1 is required for Kinesin-1 to transport cargo and to maximize its motility. Development 137, 2763–2772 10.1242/dev.048108 20630947 PMC2910386

[b55] MacDougall N. Clark A. MacDougall E. Davis I. (2003). Drosophila gurken (TGFalpha) mRNA localizes as particles that move within the oocyte in two dynein-dependent steps. Dev. Cell 4, 307–319 10.1016/S1534--5807(03)00058--3 12636913

[b56] Mach J. M. Lehmann R. (1997). An Egalitarian-BicaudalD complex is essential for oocyte specification and axis determination in Drosophila. Genes Dev. 11, 423–435 10.1101/gad.11.4.423 9042857

[b57] Marchand V. Gaspar I. Ephrussi A. (2012). An intracellular transmission control protocol: assembly and transport of ribonucleoprotein complexes. Curr. Opin. Cell Biol. 24, 202–210 10.1016/j.ceb.2011.12.014 22278045

[b58] McDermott S. M. Meignin C. Rappsilber J. Davis I. (2012). Drosophila Syncrip binds the gurken mRNA localisation signal and regulates localised transcripts during axis specification. Biol. Open 1, 488–497 10.1242/bio.2012885 23213441 PMC3507208

[b59] Medioni C. Mowry K. Besse F. (2012). Principles and roles of mRNA localization in animal development. Development 139, 3263–3276 10.1242/dev.078626 22912410 PMC3424039

[b61] Montpetit B. Thomsen N. D. Helmke K. J. Seeliger M. A. Berger J. M. Weis K. (2011). A conserved mechanism of DEAD-box ATPase activation by nucleoporins and InsP6 in mRNA export. Nature 472, 238–242 10.1038/nature09862 21441902 PMC3078754

[b62] Mor A. Suliman S. Ben-Yishay R. Yunger S. Brody Y. Shav-Tal Y. (2010). Dynamics of single mRNP nucleocytoplasmic transport and export through the nuclear pore in living cells. Nat. Cell Biol. 12, 543–552 10.1038/ncb2056 20453848

[b63] Muddashetty R. S. Nalavadi V. C. Gross C. Yao X. Xing L. Laur O. Warren S. T. Bassell G. J. (2011). Reversible inhibition of PSD-95 mRNA translation by miR-125a, FMRP phosphorylation, and mGluR signaling. Mol. Cell 42, 673–688 10.1016/j.molcel.2011.05.006 21658607 PMC3115785

[b64] Müller M. Heym R. G. Mayer A. Kramer K. Schmid M. Cramer P. Urlaub H. Jansen R-P. Niessing D. (2011). A cytoplasmic complex mediates specific mRNA recognition and localization in yeast. PLoS Biol. 9, e1000611 10.1371/journal.pbio.1000611 21526221 PMC3079584

[b65] Nakamura A. Sato K. Hanyu-Nakamura K. (2004). Drosophila cup is an eIF4E binding protein that associates with Bruno and regulates oskar mRNA translation in oogenesis. Dev. Cell 6, 69–78 10.1016/S1534--5807(03)00400--3 14723848

[b66] Natalizio B. J. Wente S. R. (2013). Postage for the messenger: designating routes for nuclear mRNA export. Trends Cell Biol. 23, 365–373 10.1016/j.tcb.2013.03.006 23583578 PMC3729607

[b67] Navarro C. Puthalakath H. Adams J. M. Strasser A. Lehmann R. (2004). Egalitarian binds dynein light chain to establish oocyte polarity and maintain oocyte fate. Nat. Cell Biol. 6, 427–435 10.1038/ncb1122 15077115

[b68] Otero L. J. Devaux A. Standart N. (2001). A 250-nucleotide UA-rich element in the 3′ untranslated region of Xenopus laevis Vg1 mRNA represses translation both in vivo and in vitro. RNA 7, 1753–1767 11780632 PMC1370215

[b69] Park H. Y. Lim H. Yoon Y. J. Follenzi A. Nwokafor C. Lopez-Jones M. Meng X. Singer R. H. (2014). Visualization of dynamics of single endogenous mRNA labeled in live mouse. Science 343, 422–424 10.1126/science.1239200 24458643 PMC4111226

[b70] Parton R. M. Hamilton R. S. Ball G. Yang L. Cullen C. F. Lu W. Ohkura H. Davis I. (2011). A PAR-1-dependent orientation gradient of dynamic microtubules directs posterior cargo transport in the Drosophila oocyte. J. Cell Biol. 194, 121–135 10.1083/jcb.201103160 21746854 PMC3135408

[b71] Patel V. L. Mitra S. Harris R. Buxbaum A. R. Lionnet T. Brenowitz M. Girvin M. Levy M. Almo S. C. Singer R. H. (2012). Spatial arrangement of an RNA zipcode identifies mRNAs under post-transcriptional control. Genes Dev. 26, 43–53 10.1101/gad.177428.111 22215810 PMC3258965

[b72] Piccioni F. Zappavigna V. Verrotti A. C. (2005). A cup full of functions. RNA Biol. 2, 125–128 10.4161/rna.2.4.2416 17114932

[b73] Pinder B. D. Smibert C. A. (2013). microRNA-independent recruitment of Argonaute 1 to nanos mRNA through the Smaug RNA-binding protein. EMBO Rep. 14, 80–86 10.1038/embor.2012.192 23184089 PMC3537145

[b74] Politz J. C. R. Tuft R. A. Pederson T. (2003). Diffusion-based transport of nascent ribosomes in the nucleus. Mol. Biol. Cell 14, 4805–4812 10.1091/mbc.E03--06--0395 12960421 PMC284785

[b75] Powrie E. A. Zenklusen D. Singer R. H. (2011). A nucleoporin, Nup60p, affects the nuclear and cytoplasmic localization of ASH1 mRNA in S. cerevisiae. RNA 17, 134–144 10.1261/rna.1210411 21036941 PMC3004054

[b76] Radford H. E. Meijer H. A. de Moor C. H. (2008). Translational control by cytoplasmic polyadenylation in Xenopus oocytes. Biochim. Biophys. Acta 1779, 217–229 18316045 10.1016/j.bbagrm.2008.02.002 PMC2323027

[b77] Rodríguez-Navarro S. Hurt E. (2011). Linking gene regulation to mRNA production and export. Curr. Opin. Cell Biol. 23, 302–309 10.1016/j.ceb.2010.12.002 21227675

[b78] Rosenthal E. T. Tansey T. R. Ruderman J. V. (1983). Sequence-specific adenylations and deadenylations accompany changes in the translation of maternal messenger RNA after fertilization of Spisula oocytes. J. Mol. Biol. 166, 309–327 10.1016/S0022--2836(83)80087--4 6854649

[b79] Santangelo P. J. Lifland A. W. Curt P. Sasaki Y. Bassell G. J. Lindquist M. E. Crowe J. E. Jr (2009). Single molecule-sensitive probes for imaging RNA in live cells. Nat. Methods 6, 347–349 10.1038/nmeth.1316 19349979 PMC4297622

[b80] Santini E. Huynh T. N. MacAskill A. F. Carter A. G. Pierre P. Ruggero D. Kaphzan H. Klann E. (2013). Exaggerated translation causes synaptic and behavioural aberrations associated with autism. Nature 493, 411–415 10.1038/nature11782 23263185 PMC3548017

[b81] Saulière J. Murigneux V. Wang Z. Marquenet E. Barbosa I. Le Tonquèze O. Audic Y. Paillard L. Roest Crollius H. Le Hir H. (2012). CLIP-seq of eIF4AIII reveals transcriptome-wide mapping of the human exon junction complex. Nat. Struct. Mol. Biol. 19, 1124–1131 10.1038/nsmb.2420 23085716

[b82] Schuh M. (2011). An actin-dependent mechanism for long-range vesicle transport. Nat. Cell Biol. 13, 1431–1436 10.1038/ncb2353 21983562 PMC3783939

[b83] Shandilya J. Roberts S. G. E. (2012). The transcription cycle in eukaryotes: from productive initiation to RNA polymerase II recycling. Biochim. Biophys. Acta 1819, 391–400 10.1016/j.bbagrm.2012.01.010 22306664

[b84] Sharma A. Yilmaz A. Marsh K. Cochrane A. Boris-Lawrie K. (2012). Thriving under stress: selective translation of HIV-1 structural protein mRNA during Vpr-mediated impairment of eIF4E translation activity. PLoS Pathog. 8, e1002612 10.1371/journal.ppat.1002612 22457629 PMC3310836

[b85] Singh G. Kucukural A. Cenik C. Leszyk J. D. Shaffer S. A. Weng Z. Moore M. J. (2012). The cellular EJC interactome reveals higher-order mRNP structure and an EJC-SR protein nexus. Cell 151, 750–764 10.1016/j.cell.2012.10.007 23084401 PMC3522173

[b86] Sinsimer K. S. Lee J. J. Thiberge S. Y. Gavis E. R. (2013). Germ plasm anchoring is a dynamic state that requires persistent trafficking. Cell Rep. 5, 1169–1177 10.1016/j.celrep.2013.10.045 24290763 PMC4149184

[b87] Snee M. J. Arn E. A. Bullock S. L. Macdonald P. M. (2005). Recognition of the bcd mRNA localization signal in Drosophila embryos and ovaries. Mol. Cell. Biol. 25, 1501–1510 10.1128/MCB.25.4.1501--1510.2005 15684399 PMC548018

[b88] Speese S. D. Ashley J. Jokhi V. Nunnari J. Barria R. Li Y. Ataman B. Koon A. Chang Y-T. Li Q. (2012). Nuclear envelope budding enables large ribonucleoprotein particle export during synaptic Wnt signaling. Cell 149, 832–846 10.1016/j.cell.2012.03.032 22579286 PMC3371233

[b89] St Johnston D. (2005). Moving messages: the intracellular localization of mRNAs. Nat. Rev. Mol. Cell Biol. 6, 363–375 10.1038/nrm1643 15852043

[b90] St Johnston D. Driever W. Berleth T. Richstein S. Nüsslein-Volhard C. (1989). Multiple steps in the localization of bicoid RNA to the anterior pole of the Drosophila oocyte. Development 107, **Suppl.**, 13–19 2483989 10.1242/dev.107.Supplement.13

[b91] Sung H-H. Telley I. A. Papadaki P. Ephrussi A. Surrey T. Rørth P. (2008). Drosophila ensconsin promotes productive recruitment of Kinesin-1 to microtubules. Dev. Cell 15, 866–876 10.1016/j.devcel.2008.10.006 19081075

[b92] Takatori N. Kumano G. Saiga H. Nishida H. (2010). Segregation of germ layer fates by nuclear migration-dependent localization of Not mRNA. Dev. Cell 19, 589–598 10.1016/j.devcel.2010.09.003 20951349

[b93] Teixeira D. Sheth U. Valencia-Sanchez M. A. Brengues M. Parker R. (2005). Processing bodies require RNA for assembly and contain nontranslating mRNAs. RNA 11, 371–382 10.1261/rna.7258505 15703442 PMC1370727

[b94] Tran E. J. Zhou Y. Corbett A. H. Wente S. R. (2007). The DEAD-box protein Dbp5 controls mRNA export by triggering specific RNA:protein remodeling events. Mol. Cell 28, 850–859 10.1016/j.molcel.2007.09.019 18082609

[b95] Trcek T. Singer R. H. (2010). The cytoplasmic fate of an mRNP is determined cotranscriptionally: exception or rule? Genes Dev. 24, 1827–1831 10.1101/gad.1972810 20810644 PMC2932966

[b96] Trinh M. A. Kaphzan H. Wek R. C. Pierre P. Cavener D. R. Klann E. (2012). Brain-specific disruption of the eIF2α kinase PERK decreases ATF4 expression and impairs behavioral flexibility. Cell Rep. 1, 676–688 10.1016/j.celrep.2012.04.010 22813743 PMC3401382

[b97] Valkov E. Dean J. C. Jani D. Kuhlmann S. I. Stewart M. (2012). Structural basis for the assembly and disassembly of mRNA nuclear export complexes. Biochim. Biophys. Acta 1819, 578–592 10.1016/j.bbagrm.2012.02.017 22406340

[b98] Van De Bor V. Hartswood E. Jones C. Finnegan D. Davis I. (2005). gurken and the I factor retrotransposon RNAs share common localization signals and machinery. Dev. Cell 9, 51–62 10.1016/j.devcel.2005.04.012 15992540

[b99] Weil T. T. Forrest K. M. Gavis E. R. (2006). Localization of bicoid mRNA in late oocytes is maintained by continual active transport. Dev. Cell 11, 251–262 10.1016/j.devcel.2006.06.006 16890164

[b100] Weil T. T. Parton R. M. Davis I. (2010a). Making the message clear: visualizing mRNA localization. Trends Cell Biol. 20, 380–390 10.1016/j.tcb.2010.03.006 20444605 PMC2902723

[b101] Weil T. T. Xanthakis D. Parton R. Dobbie I. Rabouille C. Gavis E. R. Davis I. (2010b). Distinguishing direct from indirect roles for bicoid mRNA localization factors. Development 137, 169–176 10.1242/dev.044867 20023172 PMC2796925

[b102] Weil T. T. Parton R. M. Herpers B. Soetaert J. Veenendaal T. Xanthakis D. Dobbie I. M. Halstead J. M. Hayashi R. Rabouille C. (2012). Drosophila patterning is established by differential association of mRNAs with P bodies. Nat. Cell Biol. 14, 1305–1315 10.1038/ncb2627 23178881 PMC4066581

[b103] Wilhelm J. E. Vale R. D. (1993). RNA on the move: the mRNA localization pathway. J. Cell Biol. 123, 269–274 10.1083/jcb.123.2.269 8408211 PMC2119838

[b104] Wilhelm J. E. Hilton M. Amos Q. Henzel W. J. (2003). Cup is an eIF4E binding protein required for both the translational repression of oskar and the recruitment of Barentsz. J. Cell Biol. 163, 1197–1204 10.1083/jcb.200309088 14691132 PMC2173729

[b105] Wilkie G. S. Davis I. (2001). Drosophila wingless and pair-rule transcripts localize apically by dynein-mediated transport of RNA particles. Cell 105, 209–219 10.1016/S0092--8674(01)00312--9 11336671

[b106] Wu B. Chao J. A. Singer R. H. (2012). Fluorescence fluctuation spectroscopy enables quantitative imaging of single mRNAs in living cells. Biophys. J. 102, 2936–2944 10.1016/j.bpj.2012.05.017 22735544 PMC3379624

[b107] Xu D. Shen W. Guo R. Xue Y. Peng W. Sima J. Yang J. Sharov A. Srikantan S. Yang J. (2013). Top3β is an RNA topoisomerase that works with fragile X syndrome protein to promote synapse formation. Nat. Neurosci. 16, 1238–1247 10.1038/nn.3479 23912945 PMC3853347

[b108] Yisraeli J. K. Sokol S. Melton D. A. (1990). A two-step model for the localization of maternal mRNA in Xenopus oocytes: involvement of microtubules and microfilaments in the translocation and anchoring of Vg1 mRNA. Development 108, 289–298 2351071 10.1242/dev.108.2.289

[b109] Yunger S. Rosenfeld L. Garini Y. Shav-Tal Y. (2013). Quantifying the transcriptional output of single alleles in single living mammalian cells. Nat. Protoc. 8, 393–408 10.1038/nprot.2013.008 23424748 PMC3597184

[b110] Zimyanin V. L. Belaya K. Pecreaux J. Gilchrist M. J. Clark A. Davis I. St Johnston D. (2008). In vivo imaging of oskar mRNA transport reveals the mechanism of posterior localization. Cell 134, 843–853 10.1016/j.cell.2008.06.053 18775316 PMC2585615

