# Supplementary material for: Subcellular mRNA localisation at a glance
Source: J Cell Sci. 2014 May 15;127(10):2127–33. doi: 10.1242/jcs.114272 (PMC4021467; doi:10.1242/jcs.114272)
Supplement: Poster Panels [file supp_127_10_2127__index.html]

Subcellular mRNA localisation at a glance — Poster Panels 

# Subcellular mRNA localisation at a glance

## JCS114272 Poster Panels

**Files in this Data Supplement:**

- **Poster Panel 1** - **Nuclear export of mRNAs.**
- **Poster Panel 2** - **Formation of mRNA particles.**
- **Poster Panel 3** - **Transport of mRNA particles.**
- **Poster Panel 4** - **Anchoring of mRNA particles.**
- **Poster Panel 5** - **mRNA translation.**
- **Poster Panel 6** - **mRNA degradation.**
