# Supplementary figures and images for: Subcellular mRNA localisation at a glance
Source: J Cell Sci. 2014 May 15;127(10):2127–33. doi: 10.1242/jcs.114272 (PMC4021467; doi:10.1242/jcs.114272)

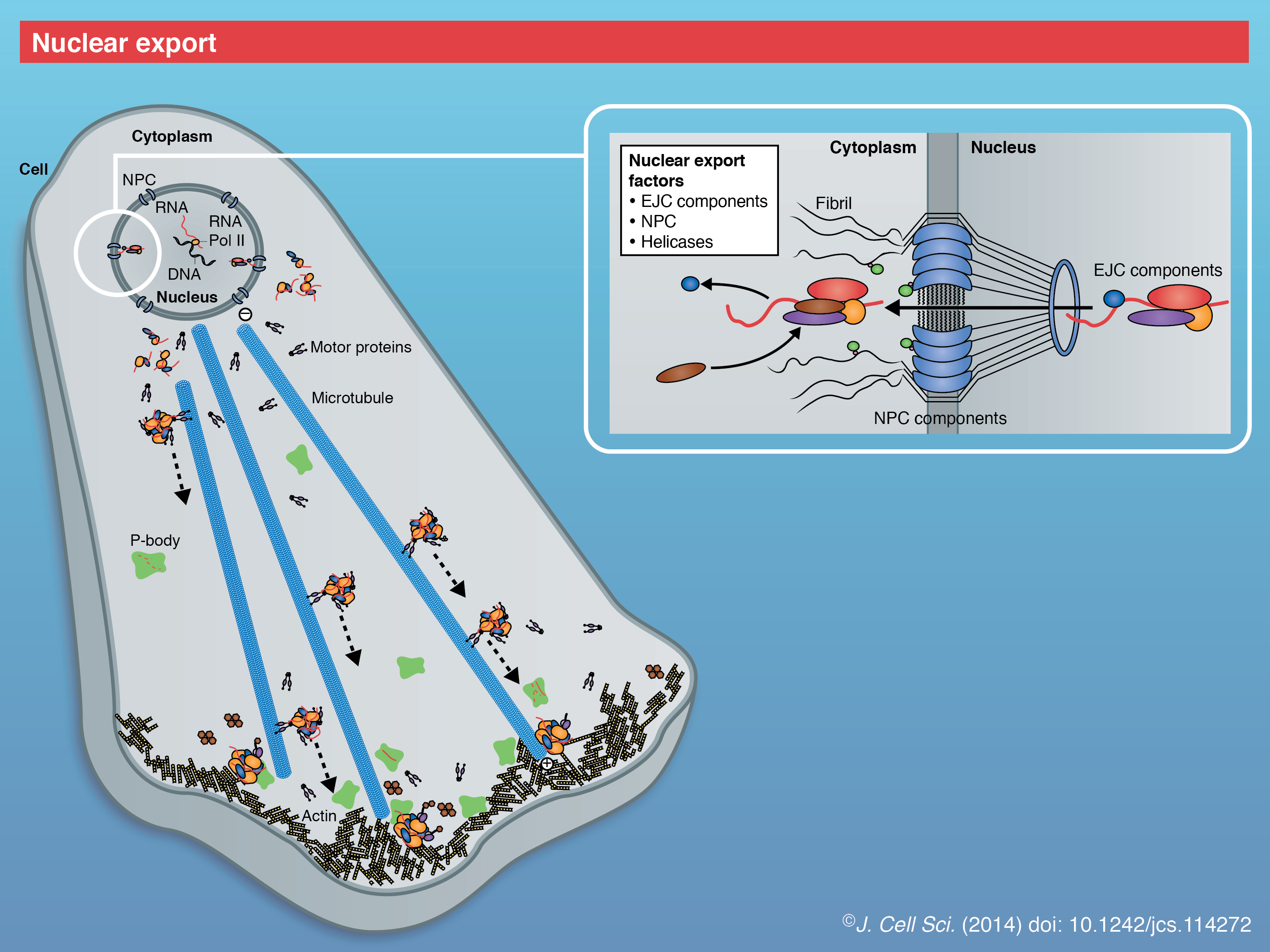

Supplement: Poster Panels [file supp_127.10.2127_JCS114272Panel1.jpg]

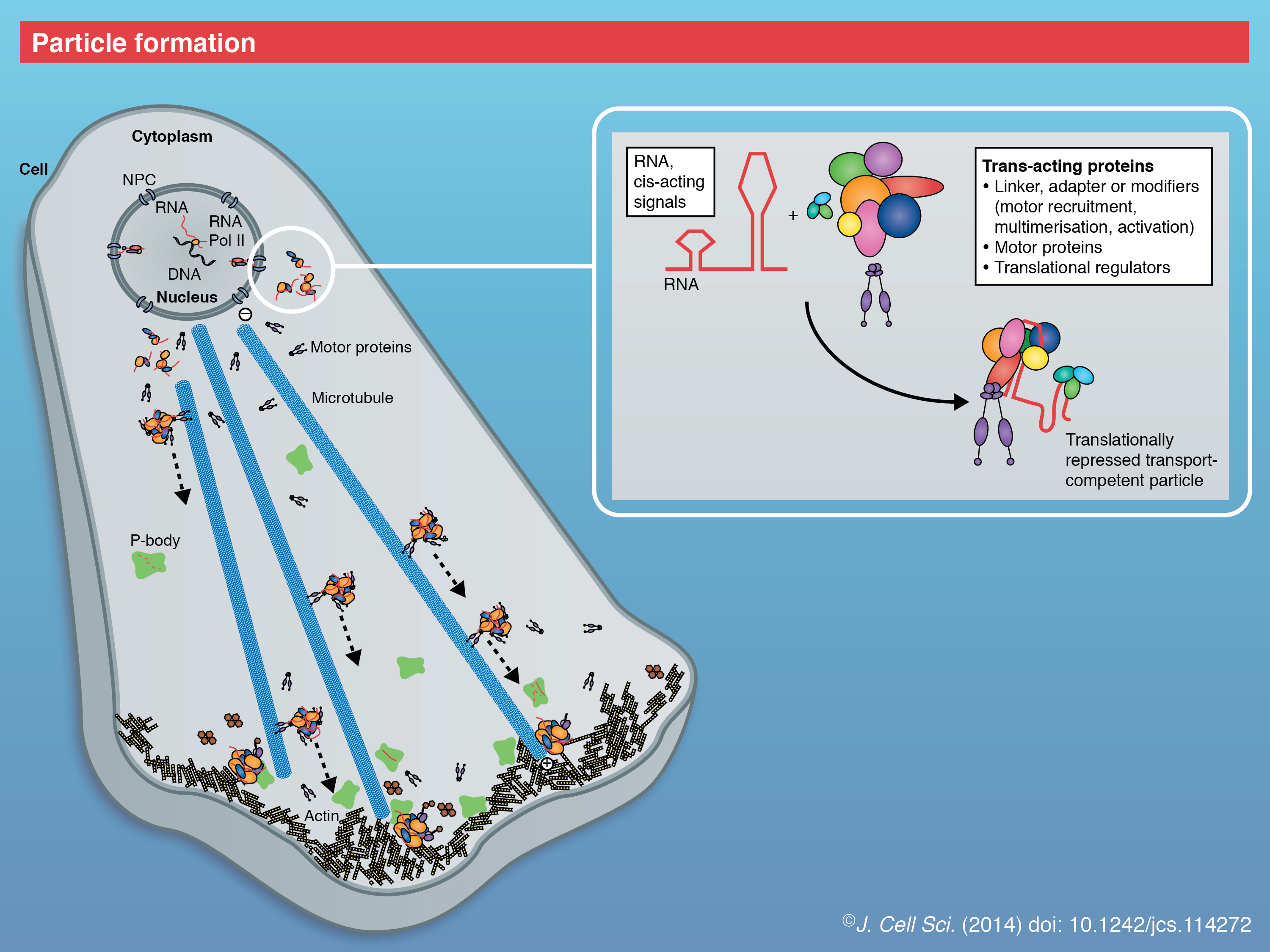

Supplement: Poster Panels [file supp_127.10.2127_JCS114272Panel2.jpg]

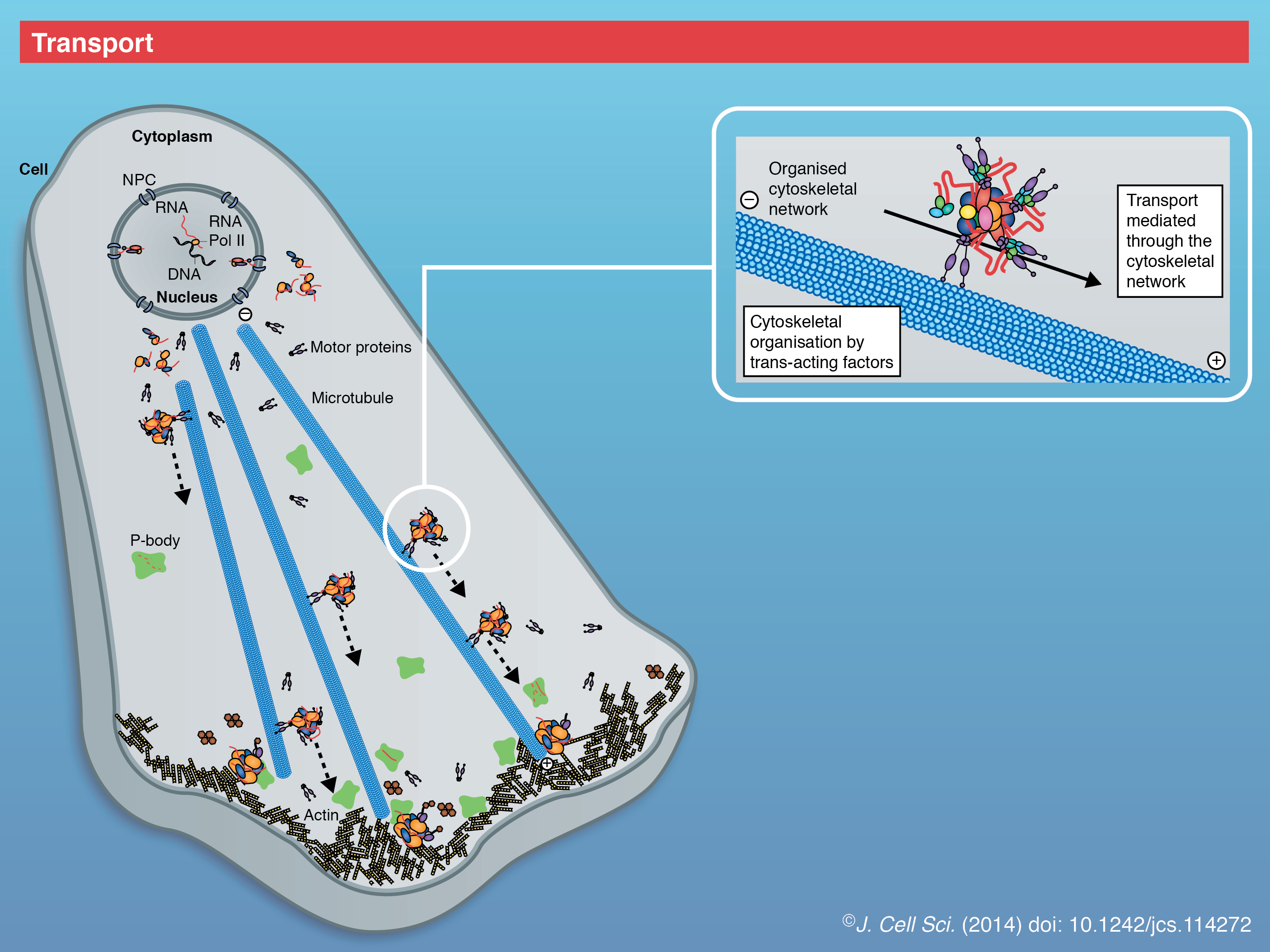

Supplement: Poster Panels [file supp_127.10.2127_JCS114272Panel3.jpg]

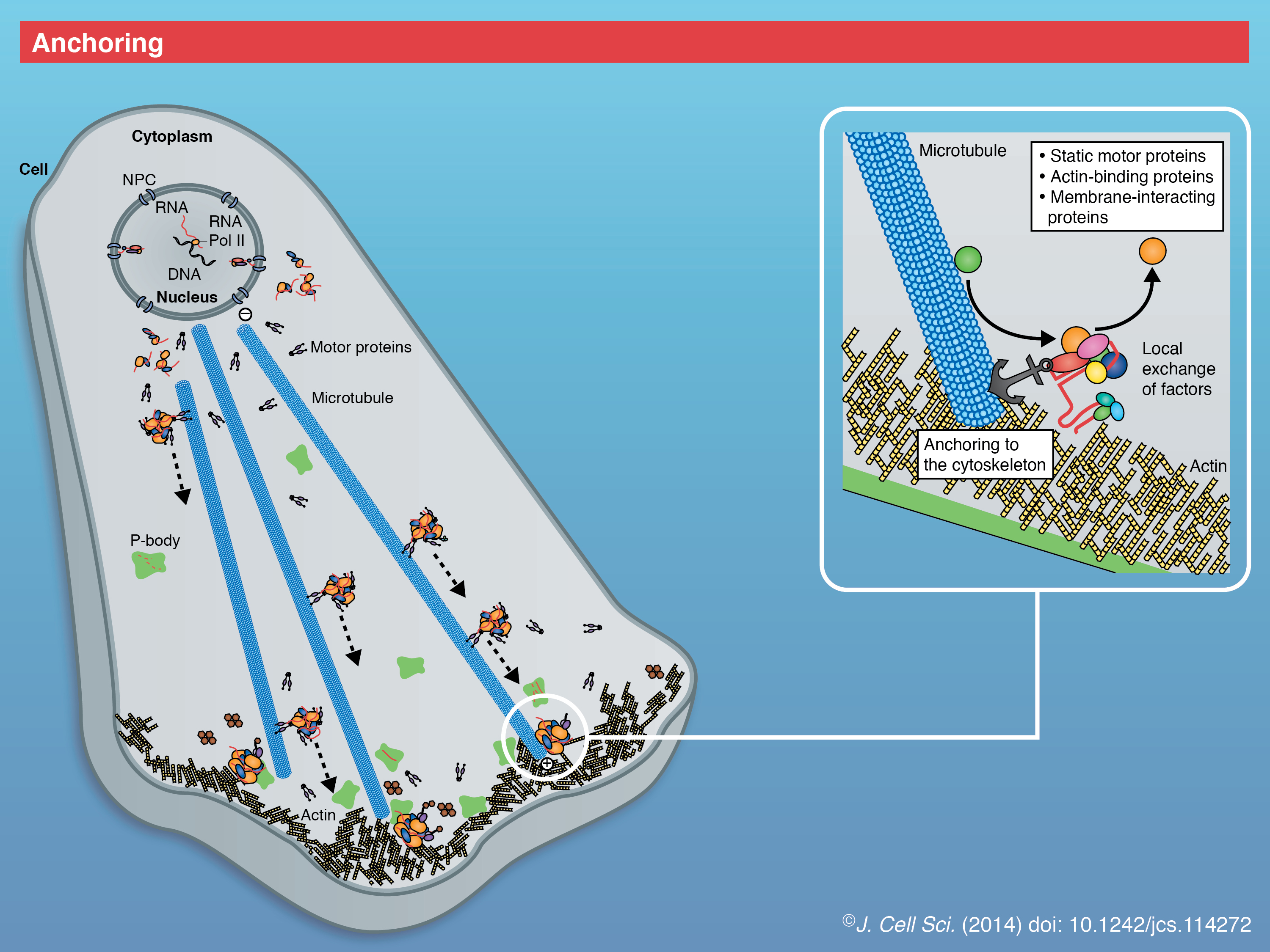

Supplement: Poster Panels [file supp_127.10.2127_JCS114272Panel4.jpg]

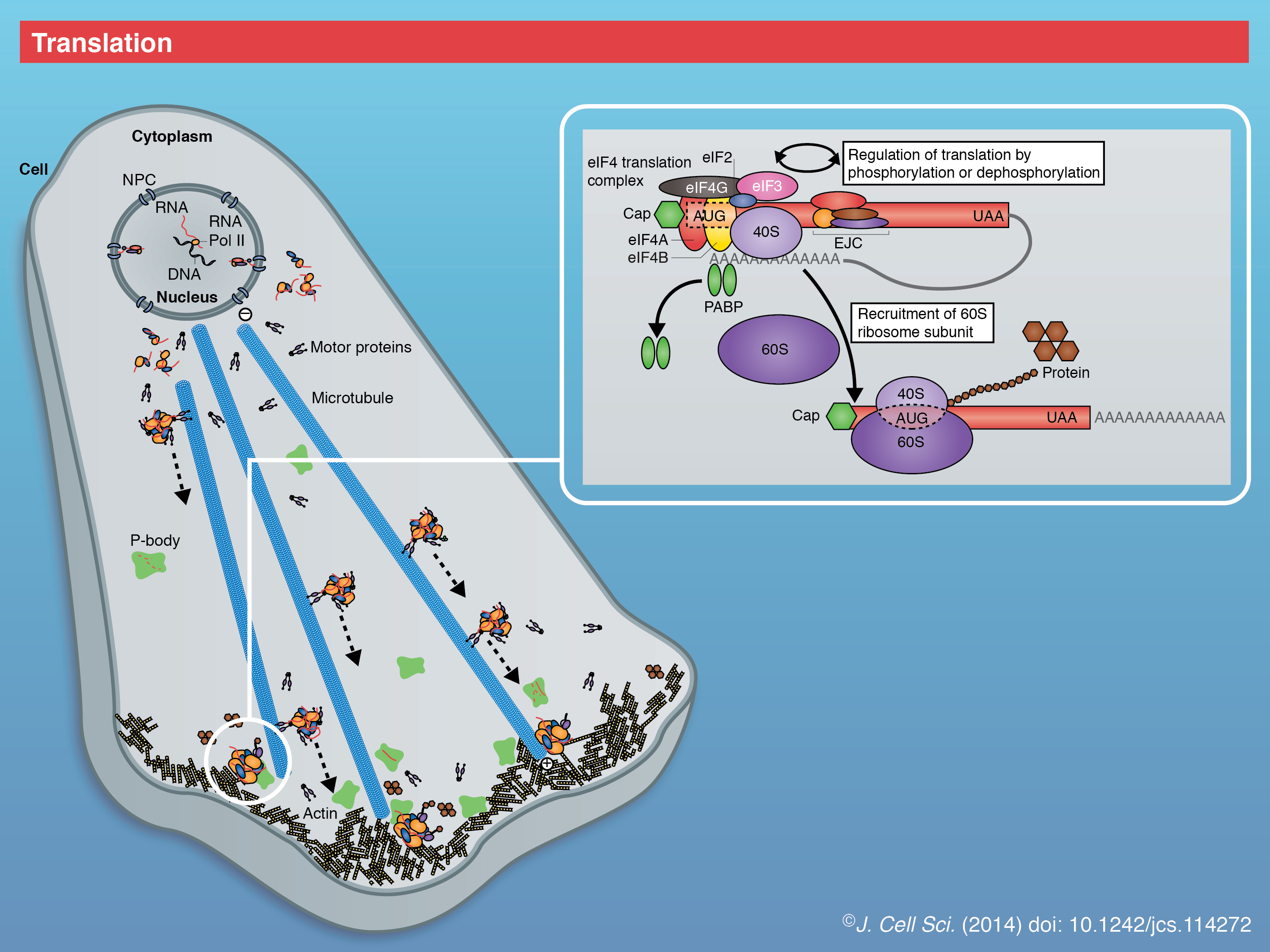

Supplement: Poster Panels [file supp_127.10.2127_JCS114272Panel5.jpg]

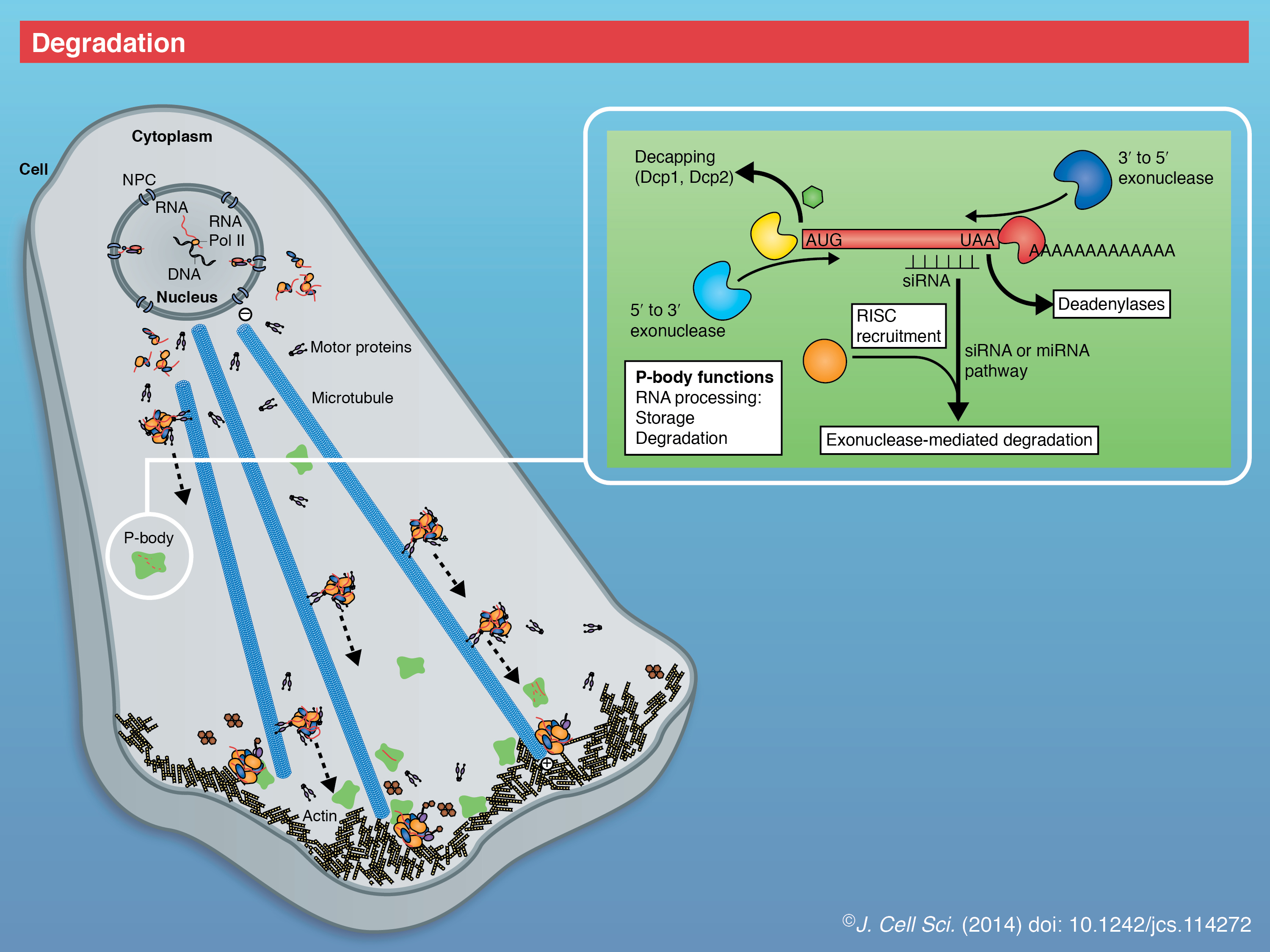

Supplement: Poster Panels [file supp_127.10.2127_JCS114272Panel6.jpg]
